# DFPS: An Efficient Downsampling Algorithm Designed for the Global Feature Preservation of Large-Scale Point Cloud Data

**DOI:** 10.3390/s25144279

**Published:** 2025-07-09

**Authors:** Jiahui Dong, Maoyi Tian, Jiayong Yu, Guoyu Li, Yunfei Wang, Yuxin Su

**Affiliations:** 1 College of Geodesy and Geomatics, Shandong University of Science and Technology, Qingdao 266590, China; 202383020007@sdust.edu.cn (J.D.); 202382020080@sdust.edu.cn (Y.W.); syx041021@126.com (Y.S.); 2 School of Civil Engineering, Anhui Jianzhu University, Hefei 230601, China; 3 Qingdao Xiushan Mobile Survey Co., Ltd., Qingdao 266510, China; liguoyuaa@163.com

**Keywords:** large-scale point cloud processing, downsampling, feature preservation

## Abstract

This paper introduces an efficient 3D point cloud downsampling algorithm (DFPS) based on adaptive multi-level grid partitioning. By leveraging an adaptive hierarchical grid partitioning mechanism, the algorithm dynamically adjusts computational intensity in accordance with terrain complexity. This approach effectively balances the global feature retention of point cloud data with computational efficiency, making it highly adaptable to the growing trend of large-scale 3D point cloud datasets. DFPS is designed with a multithreaded parallel acceleration architecture, which significantly enhances processing speed. Experimental results demonstrate that, for a point cloud dataset containing millions of points, DFPS reduces processing time from approximately 161,665 s using the original FPS method to approximately 71.64 s at a 12.5% sampling rate, achieving an efficiency improvement of over 2200 times. As the sampling rate decreases, the performance advantage becomes more pronounced: at a 3.125% sampling rate, the efficiency improves by nearly 10,000 times. By employing visual observation and quantitative analysis (with the chamfer distance as the measurement index), it is evident that DFPS can effectively preserve global feature information. Notably, DFPS does not depend on GPU-based heterogeneous computing, enabling seamless deployment in resource-constrained environments such as airborne and mobile devices, which makes DFPS an effective and lightweighting tool for providing high-quality input data for subsequent algorithms, including point cloud registration and semantic segmentation.

## 1. Introduction

With the rapid advancement of 3D perception technology, point cloud data have become a critical data modality across various domains such as geographic information systems, autonomous driving, robot navigation, and augmented reality. A key application lies in autonomous driving, where point clouds facilitate road detection, obstacle recognition, and pedestrian tracking, thereby playing an essential role in environmental perception and positioning [1,2,3,4,5]. Furthermore, 3D point clouds are widely utilized in intelligent manufacturing, modern medicine, art design, and cultural heritage preservation, highlighting their broad applicability across disciplines [6,7,8].

Point cloud data are primarily acquired through LiDAR (Light Detection and Ranging) sensors and multi-view photogrammetry techniques. These methods offer unmatched precision in capturing surface morphology and object characteristics [9]. Structurally, point clouds are represented as multi-dimensional attribute matrices and are commonly stored in formats such as .bin, .pcd, .ply, .txt, and .csv [10,11]. The evolution of 3D perception technology has resulted in an exponential increase in the volume of point cloud data. High-precision multi-beam LiDAR systems can now capture millions of points per frame by significantly enhancing angular resolution and scanning frequency [12]. Advances in solid-state LiDAR, particularly in channel integration and signal processing, have substantially improved the capacity of automotive perception systems to generate dense point clouds within short time intervals [3,13,14,15,16]. This provides detailed information for scene reconstruction, enabling the detection and quantification of road defects in point cloud data, as well as supporting applications that require large-scale point cloud datasets—such as large-scale bridge construction—by delivering high-quality, dense point clouds tailored to these demanding tasks [17,18]. However, in data processing workflows for applications like autonomous driving and large-scale point cloud Level of Detail (LOD) visualization [19], the sheer volume of point cloud data presents significant challenges in registration, segmentation, and visualization [6,20,21,22]. The surge in data volume leads to non-linear increases in computational complexity, imposing significant costs on storage and transmission [23,24,25]. Under these circumstances, point cloud downsampling has emerged as a vital preprocessing technique, offering three primary advantages:

1. Reduction of computational complexity: Large volumes of point cloud data present significant challenges to point cloud processing algorithms. Directly processing massive point clouds can impose a substantial computational burden. Downsampling mitigates this issue by reducing the number of points through predefined sampling rules and logic, thereby effectively lowering the computational load in subsequent processing stages [11,26].
2. Improvement of data quality: Appropriate downsampling algorithms can effectively address inconsistencies in point density that arise during raw data acquisition due to factors such as equipment limitations and environmental conditions. These inconsistencies can negatively affect downstream processing tasks. For instance, in point cloud registration, varying point densities may lead to poor alignment between point sets [27,28]. Similarly, in aerial surveying, spliced point clouds often contain numerous redundant points caused by longitudinal and lateral overlaps, which can degrade both processing speed and modeling accuracy [29,30].
3. Integration of deep learning: With the rapid development of deep learning, various models for point cloud registration, classification, and segmentation have emerged. Notable examples include Qi’s PointNet [31] and PointNet++ [26], Li’s PointCNN [32], and Yang’s RITNet [4], which serve as foundational architectures for point cloud data processing. In deep-learning applications, downsampling techniques based on the farthest-point sampling algorithm have gained widespread adoption due to their ability to preserve global features and standardize input formats [33]. These techniques enable point clouds to meet specific network input requirements—such as the standardized 2048-point input required by the PointNet series—while retaining critical feature information.

Current point cloud downsampling methods can be categorized into three primary approaches: rule-based sampling, deep-learning-based sampling, and geometric feature-based sampling. Firstly, the rule-based downsampling methods are regarded as the most convenient methods, including random sampling, uniform sampling, and voxel-based sampling, which prioritize computational simplicity and efficiency. However, they exhibit notable limitations, such as edge detail degradation and inadequate accuracy for complex or high-precision applications [16,34,35]. Secondly, the deep-learning-based downsampling methods face several technical challenges in resource-constrained engineering environments, including dependency on large labeled datasets for training, resulting in high memory consumption and computational overhead, which hinders real-time processing capabilities [34]. High costs are associated with data acquisition and annotation, often leading to performance degradation and poor generalization in data-scarce or cross-domain scenarios due to overfitting. Thirdly, the geometric feature-based downsampling strategies, such as farthest-point sampling (FPS) and curvature-aware sampling, offer distinct advantages. FPS, in particular, has become a standard preprocessing module in many prominent deep-learning models (e.g., PointCNN, PointNet++) [20,36,37,38,39] due to its uniform spatial coverage and topological preservation properties. However, the geometric feature-based dowsampling strategies often encounter significant computational burdens, and their low operational efficiency severely hinders their application in practical engineering. Although the FPS algorithm is highly authoritative in terms of preserving global features, it suffers from its greedy iterative distance calculation, resulting in exponentially increasing complexity with larger point clouds, creating severe performance bottlenecks in CPU-based implementations [34,37]. In high-precision applications, FPS typically consumes 30–70% of a neural network’s runtime, with this proportion scaling with data size [3,40,41]. While there are some GPU-based optimizations to improve efficiency, they lack universality and remain unsuitable for resource-constrained environments. In many practical engineering applications, the feasibility of lightweight deployment is of great significance.

Based on the aforementioned analysis, this paper presents DFPS, an efficient downsampling algorithm designed for the global feature preservation of large-scale point cloud data. The proposed DFPS algorithm enhances conventional sampling methodology through two key innovations: an adaptive grid partitioning mechanism that optimizes spatial sampling logic, and a multithreaded parallel computing architecture that accelerates processing throughput. Notably, the algorithm’s lightweight design ensures GPU-independent operation, making it particularly suitable for resource-constrained engineering applications.

The remaining part of this article is arranged as follows:

Section 2 elaborates on the DFPS algorithmic framework, including its operational workflow and fundamental principles.

Section 3 presents comprehensive experimental validation, featuring comparative analyses of sampling efficiency and quality assessment, which collectively demonstrate DFPS’s superior performance as a 3D point cloud downsampling solution.

Section 4 concludes with a discussion of DFPS’s distinctive characteristics, applicable domains, and potential engineering applications.

## 2. Methodology

**Figure 1:**
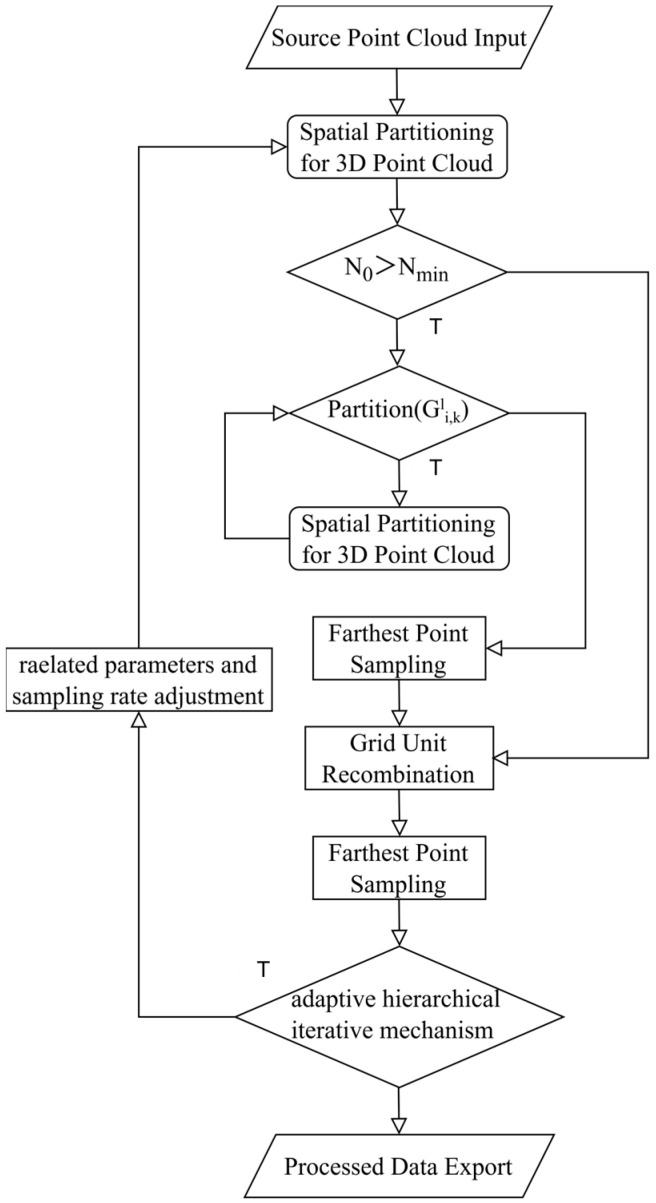
Flowchart of the DFPS algorithm, showing the specific logic of adaptive hierarchical grid partitioning.

The DFPS algorithm employs an adaptive hierarchical grid partitioning mechanism for iterative farthest-point sampling. This mechanism systematically incorporates critical factors, including the hardware performance of the experimental equipment, the scale of the point cloud data, and the diverse processing objectives for point clouds. Through an iterative optimization process, the algorithm performs dynamic grid partitioning, thereby achieving a rational and efficient multi-level grid division of the entire point cloud dataset. The adaptive hierarchical iterative mechanism can be used to control the number of point cloud outputs to match the preset sampling rate. A distinctive feature of DFPS is its adjustable parameter β, which enables manual calibration of the weight assigned to preserving local details, catering to varying requirements in point cloud data processing tasks. Apart from the adaptive hierarchical grid partitioning mechanism, the algorithm incorporates a multithreaded parallel acceleration architecture, ensuring computational efficiency during the downsampling process of point clouds. The comprehensive logical framework of the DFPS algorithm is illustrated in Figure 1, demonstrating its systematic workflow.

As illustrated in Figure 1, the sampling process adopts an iterative farthest-point sampling approach to ensure both processing efficiency and sampling accuracy. The implementation follows these steps: Firstly, the raw data undergo spatial partitioning into eight equal segments. The system then evaluates whether the initial point cloud count N_0_ exceeds N_min_, where N_min_ represents the predetermined threshold for initiating the adaptive hierarchical grid partitioning mechanism. This threshold is typically set to a relatively small value (such as 256) to prevent the DFPS adaptive hierarchical grid partitioning mechanism from reducing processing speed when handling small-scale point clouds. If N_0_ ≤ N_min_, the system performs grid merging followed by immediate grid recombination. If N_0_ > N_min_, the process proceeds with the adaptive hierarchical grid partitioning mechanism. This mechanism combines first-round farthest-point sampling to enable dynamic adjustment of local detail weights while significantly reducing the computational load for second-round farthest-point sampling.

The adaptive hierarchical grid partitioning determination mechanism serves as the core component of the DFPS algorithm’s iterative optimization process. This determination rule, denoted as ${Partition(G}_{i,k}^{l})$, governs the adaptive partitioning logic throughout the hierarchical grid construction.

$$\;{Partition(G}_{i,k}^{l})=\left\{\begin{array}{c} True\;\;\;\;\;\;\;\;if\;N_{i,k}^{l}\geq \theta_{upper}\\ False\;\;\;\;\;\;\;\;\;\;\;\;otherwise\;\;\;\;\; \end{array}\right.$$

Here, $N_{i,k}^{l}\;$ denotes the number of point clouds within the k-th unit of the i-th partition segment at the l-th hierarchical level ($S_{i,k}^{l}$). The parameter $\theta_{upper}$ represents the point cloud partitioning threshold, defined as the minimum point cloud count required to initiate a partition. This threshold is determined immediately after loading the raw data into the system, with its calculation formula expressed as follows:

$$\;\theta_{upper}=\beta \left(\frac{L_{min}}{d\cdot L_{0}}\right)\frac{{\gamma N}_{0}}{n_{c}c_{b}}$$

Here, β serves as an adjustable parameter, typically initialized to 1, which globally modulates the partitioning threshold. For instance, when enhanced preservation of local details is required in practical applications, reducing β decreases $\theta_{upper}$, thereby lowering the partitioning threshold. γ is the parameter of the adaptive hierarchical iterative mechanism, and it is adjusted during the adaptive hierarchical iteration process. $N_{0}$ represents the total point count in the raw dataset, while $n_{c}$ denotes the CPU core count and $c_{b}$ indicates the base clock frequency of the processor. $L_{0}$ corresponds to the initial block size of the raw data, and $L_{min}$ signifies the minimum resolution threshold, implemented to prevent resource inefficiency caused by excessive partitioning in extreme cases. $L_{min}$ is quantified by the grid block dimensions after successive partitioning operations, with its determination formula expressed as:

$$\;L_{min}=\frac{1}{{\lfloor \log}_{10}N_{i}\rfloor }L_{0}$$

When the adaptive hierarchical grid partitioning condition evaluates to True, the corresponding point cloud undergoes octant segmentation. Each resulting grid partition is dynamically assessed against the point cloud partitioning threshold $\;\theta_{upper}$ until meeting the iteration termination criterion. This iterative approach enables independent first-round farthest-point sampling (at the designated sampling rate) for all grid partitions. The sampled point sets $S_{i,k}^{l}$ from each grid partition are subsequently aggregated: $S=⋃S_{i,k}^{l}$. The unified point set S then undergoes second-round farthest-point sampling, producing a 50% sampled output for the adaptive hierarchical iterative mechanism’s evaluation. This iterative framework simultaneously regulates the final output point cloud’s sampling rate. Firstly, we claim that $tgt\left(0\right)=1\;;n\to \infty \;$, let $tgt\left(n\right)=tgt\left(n-1\right)\frac{n}{n+1}$. Then, $\exists n>0,tgt\left(n\right)>sampling\;rate>tgt\left(n+1\right)$.

$$while(\;i\leq n\;)\;\;\left\{\begin{array}{c} True\;\;\;\;\;\;current\;sampling\;rate=\frac{i}{i+1}\;\\ False\;\;\;\;current\;sampling\;rate=\frac{a}{tgt(i)} \end{array}\right.$$

Here, i represents the number of iterations, n is the target number of iterations, and $tgt\left(i\right)$ is the approximation value of the sampling rate calculated in the i-th iteration, while a is the target sampling rate. At this point, the true value of the sampling rate in the i-th iteration is $\frac{i}{i+1}$. When the adaptive hierarchical iteration mechanism is determined to be true, parameter adjustments are made: for γ in $\theta_{upper}$, $\gamma =\gamma_{0}*1.05$, where $\gamma_{0}$ is the γ value after the last iteration adjustment, and for $N_{min}$, $N_{min}=N_{min0}\cdot 0.75$.

When the value is False, no parameter adjustment is performed. The sampling rate is set as $rate=\frac{a}{tgt(n)}$, the final sample is taken, and the final result is output.

**Figure 2:**
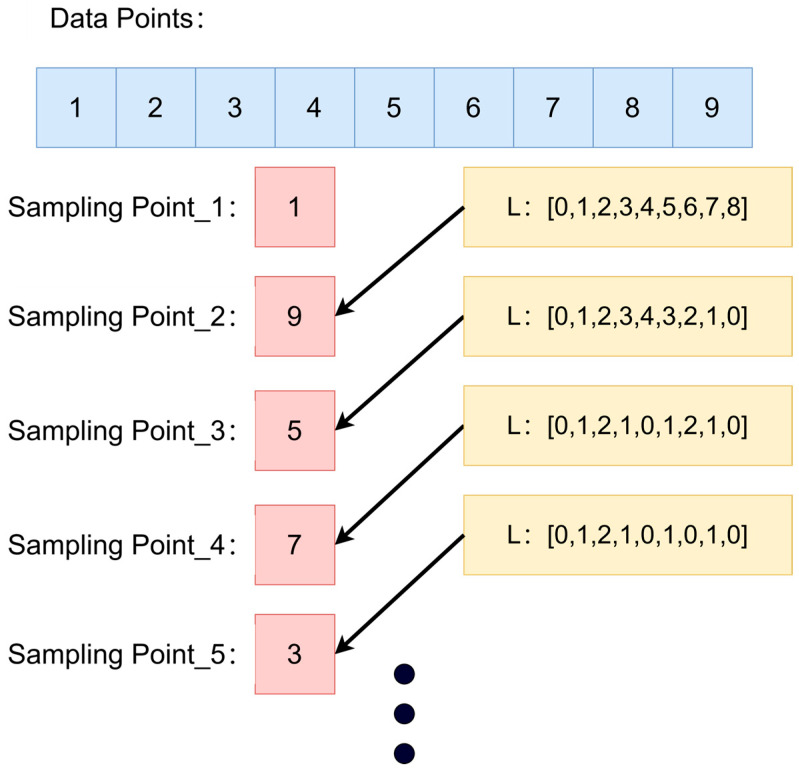
Schematic diagram of the farthest-point sampling, illustrating the basic logic of the farthest-point sampling through a simple example.

DFPS dynamically determines the maximum point count per grid cell based on the computational capabilities of the target hardware. The input point cloud is partitioned into hierarchical grids using a subdivision methodology analogous to octree structures, with a critical distinction: DFPS automatically increases partitioning depth in densely populated regions to ensure uniform point distribution across all grids, thereby maximizing efficiency gains from multithreaded parallelization. By adopting a dynamic and hierarchical downsampling method, it is more suitable for processing extremely large point cloud data, avoiding the memory usage problem caused by the logic of the farthest-point sampling itself. This strategy balances local feature preservation with global structural coherence, ensuring optimal preservation of critical feature information in the final sampled output. The core objective of farthest-point sampling is to achieve non-uniform and efficient sampling of the original point cloud by iteratively selecting the point that is farthest from the set of already sampled points. The algorithm process is described in Figure 2 with the attached diagram illustrating the principle of FPS [39]:

With a point cloud set $P=\{p_{i}\in R^{3}|i=1,...,N\}$, the iterative process of farthest-point sampling for generating a downsampled subset S⊂P is formally defined as follows:

1. Randomly select a seed point $S_{0}\subset P$ and initialize the set as $S=\{s_{0}\}$.
2. Conduct iterative sampling: for each iteration k=1,...,K−1, compute the minimum Euclidean distance from each candidate point P∈P\S to the current sampled set S: $d\left(p,S\right)=\underset{s\in S}{min}∥p-s{∥}_{2}$. Identify the candidate point with the largest minimum distance: $s_{k}=\underset{p\in P\backslash S}{argmax}d\left(p,S\right)$. Lastly, augment the sampled set: $S\leftarrow S\cup \{s_{k}\}$.

Furthermore, DFPS introduces a multithreaded parallel acceleration architecture that synergistically optimizes computational efficiency through two complementary parallelism strategies:

1. Acceleration for entire progress: This architecture parallelizes computations across adaptively partitioned grids. For instance, in the illustrated example, the initial point cloud is decomposed into eight grid blocks through one iteration of adaptive partitioning. Each grid block is assigned to an independent thread, enabling concurrent processing of localized farthest-point sampling (FPS) operations.
2. Acceleration for the single grid: Within each grid block, the algorithm further implements fine-grained parallelism during critical computational phases, such as the calculation of minimum Euclidean distances in FPS. This intra-grid parallelization exploits multi-core capabilities to accelerate distance metric evaluations and candidate point selection.

**Figure 3:**
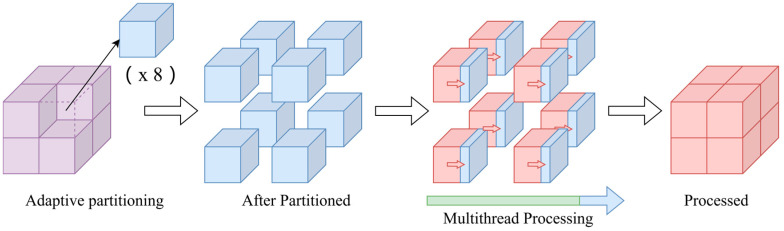
Schematic diagram of multithreaded parallel acceleration architecture.

As depicted in the Figure 3, this dual-layered acceleration mechanism operates at both the global grid decomposition level and the local per-grid computation level. The hierarchical approach minimizes computational redundancy while maximizing hardware resource utilization, particularly in scenarios involving recursive grid decomposition. By decoupling coarse-grained task parallelism from fine-grained data parallelism, DFPS achieves near-linear scalability with respect to available CPU cores, establishing a robust foundation for real-time processing of billion-scale point clouds.

## 3. Related Experiments

To validate the effectiveness and robustness of DFPS, the experiment consists of two modules. The first module focuses on verifying the operational efficiency of DFPS, while the second module is the feature preservation performance test.

### 3.1. Verification of DFPS Operational Efficiency

The operational efficiency evaluation involves comparative analysis among DFPS, standardized FPS (Farthest-Point Sampling), and FPS_After (a baseline optimized version incorporating only multithreading and point cloud data access optimization). The comparison is conducted under different sampling ratios (1/8, 1/16, 1/32) and varying point cloud scales. Synthetic spherical point clouds with uniform sizes were generated as experimental data, covering point quantities ranging from 2^9^ to 2^20^ (i.e., 512 to 1,048,576 points). These datasets represent typical processing requirements for initial point clouds in practical applications.

All tests were not conducted on high-performance computing devices, but rather on an Intel^®^ Core™ i7-9750H CPU @ 2.60 GHz platform. Three tables (Table 1, Table 2 and Table 3) present the average computation time (derived from 20 repeated executions for each point cloud scale) required by different downsampling methods to process multi-order point cloud magnitudes under specified sampling rates.

**Figure 4:**
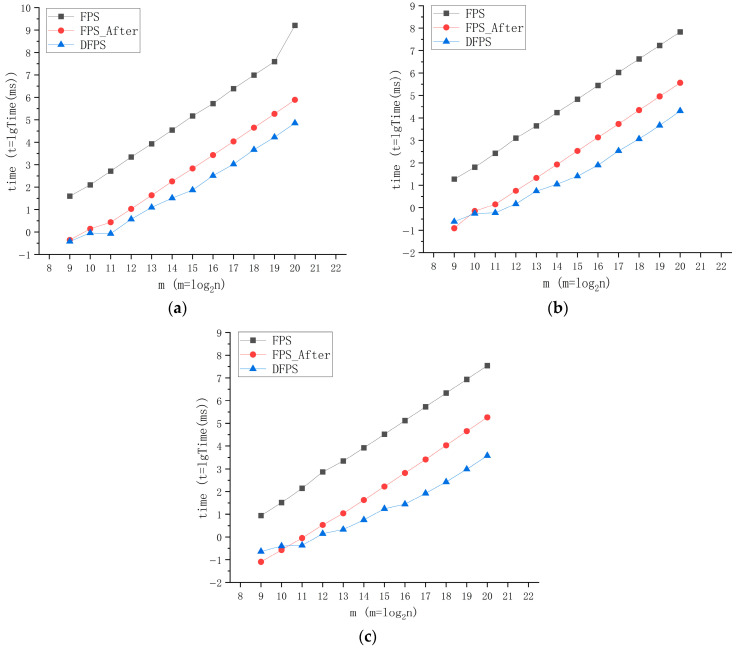
This figure shows the results of DFPS operational efficiency test, comparing three FPS methods in terms of processing efficiency at sampling rates of 12.5% (**a**), 6.25% (**b**), and 3.125% (**c**).

In order to compare the speed changes of each method more intuitively, the data in the above tables are visualized. Since the times increase exponentially with the number of points, logarithmic representation is adopted to make the effect comparison more convenient: m represents the total number of points, and Time represents the average running time required by the method for a certain number of points at this sampling rate, as shown in Figure 4.

As evidently illustrated in the preceding figure, DFPS exhibits exceptional computational efficiency. The results demonstrate that the processing speed of DFPS for point cloud data has significantly improved, particularly when handling large-scale point cloud datasets. Owing to the inherent computational logic of DFPS, while there is only a marginal improvement in the calculation performance for small-scale point cloud data, DFPS demonstrates remarkably strong processing capabilities for large-scale point cloud data. The processing delay for millions of point cloud data has been reduced from approximately 161,665 s using the original FPS method to approximately 71.64 s with DFPS (12.5% sampling rate), representing an efficiency improvement of over 2200 times. As the sampling rate requirement decreases, this improvement becomes even more pronounced: the processing time is reduced from approximately 35,060 s with the original FPS method to approximately 3.78 s with DFPS (3.125% sampling rate), achieving an efficiency improvement of nearly ten thousand times.

**Figure 5:**
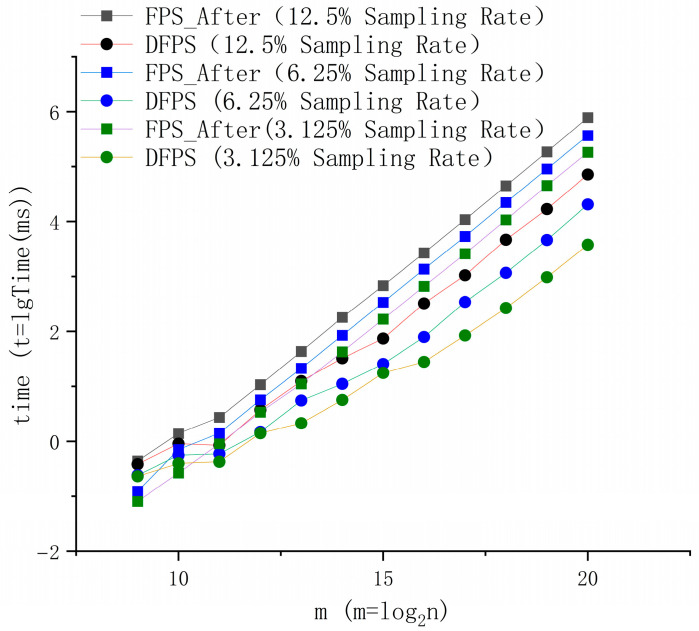
Comparison of optional efficiency of DFPS and FPS_After at different sampling rates.

In the ablation study presented in Figure 5, the performance of the adaptive hierarchical grid partitioning mechanism in managing point cloud data is evaluated. For smaller point clouds, due to unavoidable inherent computational overheads such as hierarchical grid calculations and decomposition, the speed advantage of the adaptive hierarchical grid partitioning sampling logic employed by DFPS is not significant. However, as the volume of the point cloud increases and the target sampling rate decreases, the computational superiority of DFPS’s adaptive farthest-point downsampling becomes increasingly prominent. Compared to the FPS variant (FPS_After) that solely employs a multithreaded parallel acceleration architecture, the farthest-point downsampling method based on an adaptive hierarchical grid partitioning mechanism achieves tens of times better performance, with this performance gap widening substantially as the point cloud scale expands and the sampling rate diminishes. At a sampling rate of 3.125%, the performance enhancement for a million-point cloud dataset reaches nearly 60 times. This underscores the exceptional scalability and efficiency of the adaptive hierarchical grid partitioning mechanism in large-scale processing scenarios. These findings ultimately confirm the substantial speed advantage of DFPS over traditional farthest-point sampling methods, especially in applications demanding real-time processing of large-scale point clouds.

This divergence in performance trajectories conclusively validates the effectiveness of the adaptive hierarchical grid partitioning architecture in balancing algorithmic efficiency with scalability. The trend underscores two critical insights:

1. The adaptive hierarchical grid partitioning mechanism achieves optimal resource utilization only when the point cloud scale and sampling complexity surpass a critical threshold, beyond which its hierarchical parallelism significantly outperforms flat multithreaded implementations. Considering the difference is only at the level of 10-1 milliseconds, this issue can be disregarded in the vast majority of engineering applications. If the task has certain requirements for timeliness, a judgment mechanism can be added subsequently.
2. The algorithmic advantage amplifies inversely with sampling rate, demonstrating DFPS’s capability to maintain sublinear time complexity under aggressive downsampling requirements—a capability absent in conventional FPS variants, which affirms that the adaptive hierarchical grid partitioning mechanism is not merely an auxiliary optimization but a fundamental enabler of scalable, high-performance point cloud processing.

### 3.2. DFPS Feature Preservation Performance Test

Lightweight processing of large-scale point cloud data is a critical preprocessing step in point cloud data analysis. In most point cloud processing algorithms, high-quality input data significantly influence the processing outcome, and effective point cloud data downsampling is essential. The farthest-point sampling (FPS) algorithm is widely acknowledged for its superior feature retention capabilities. However, whether the DFPS algorithm with an adaptive hierarchical grid partitioning mechanism can achieve comparable global feature retention to the FPS algorithm remains to be experimentally validated. This module evaluates the global feature retention capability through visual observation and comparison. To intuitively demonstrate the sampling quality of DFPS and validate its effectiveness, a test module is designed using large-scale airborne point cloud data, which include both large-scale airborne marine survey data and large-scale airborne land survey data.

#### 3.2.1. Experiments on Marine Survey Data

The marine survey data were acquired by the MAPPER-20kU unmanned aerial vehicle-mounted water and land detection laser radar developed by the Shanghai Institute of Optics and Fine Mechanics, Chinese Academy of Sciences, China. This system conducted on-site airborne blue–green laser radar measurements in Jiuzhaigou, China, as well as in a shallow sea region in Danzhou City, Hainan Province, China. Table 4 provides detailed parameter information for the MAPPER-20kU unmanned aerial vehicle-mounted water and land detection laser radar.

**Figure 6:**
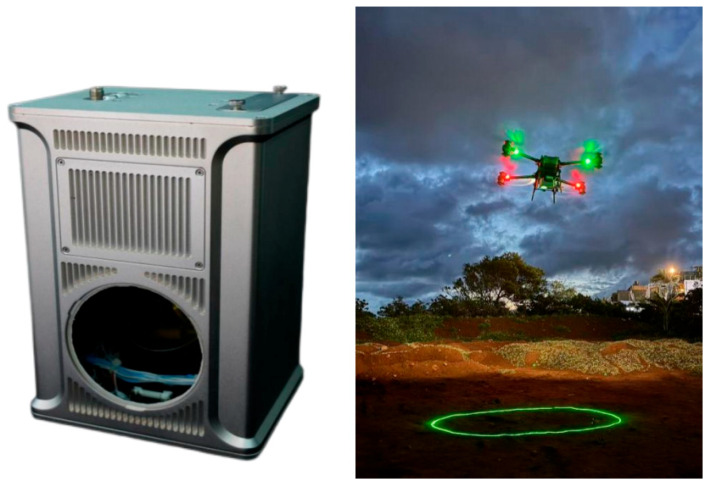
Unmanned aerial vehicle-mounted water and land detection laser radar MAPPER-20kU product diagram.

Figure 6 shows the comparison effect of global and local visual observation results obtained by downsampling the airborne blue–green laser bathymetric data of Wuhua Sea (containing 6,091,468 points) and the airborne blue–green laser bathymetric data of the shallow sea area of Danzhou (a partial view) (containing 8,261,654 points) through the DFPS algorithm with a 2% sampling rate.

**Figure 7:**
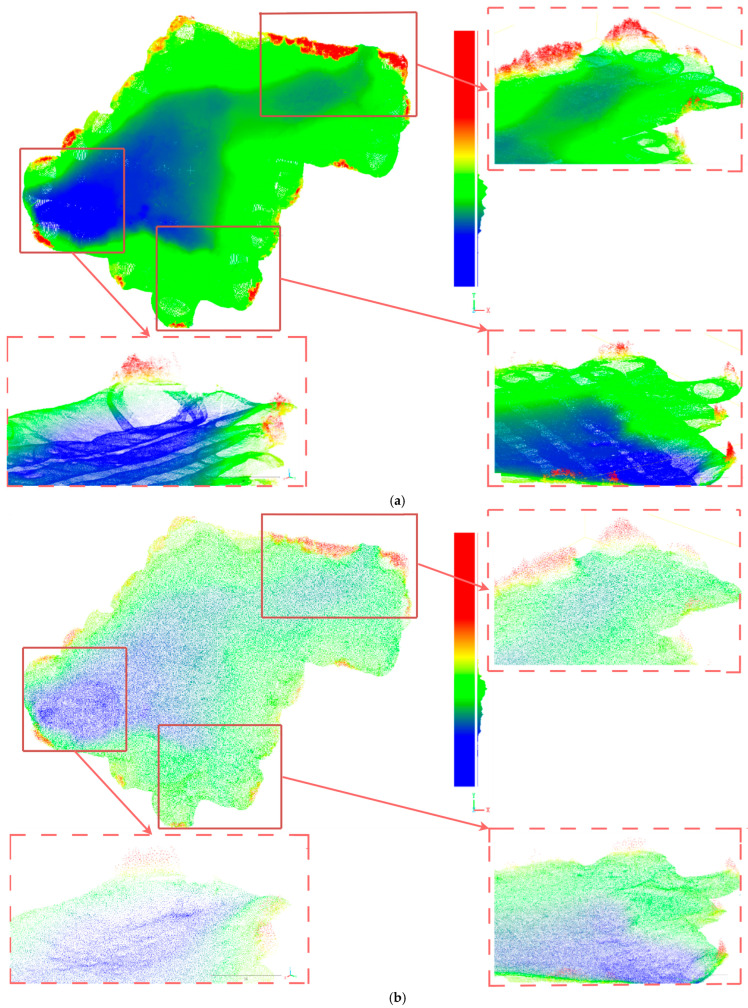

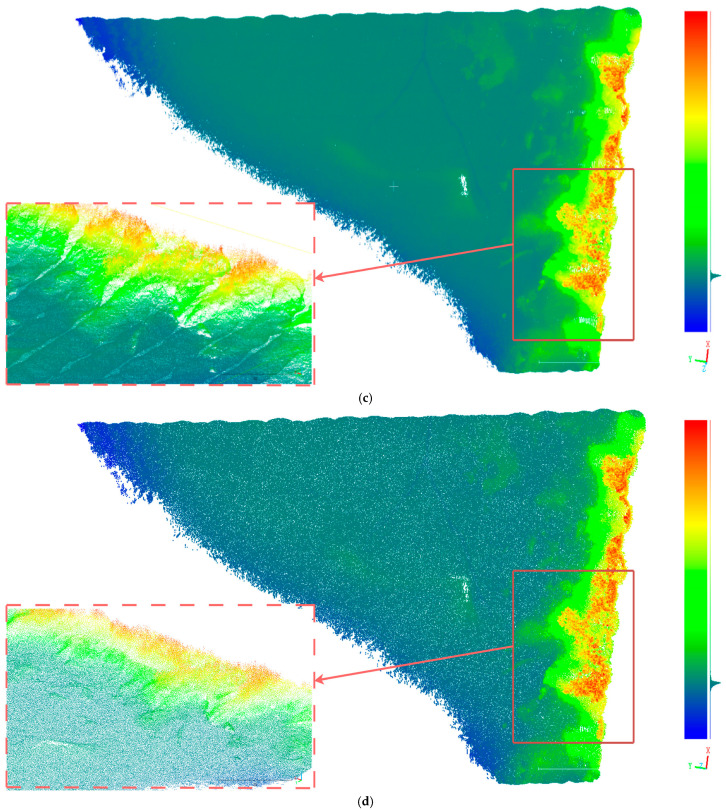
(**a**) shows the visual effect diagram of the airborne bathymetric data of Wanhua Sea before processing. (**b**) shows the visual diagram of the airborne bathymetric data of Wanhua Sea after processing. (**c**) shows the visual effect diagram of the airborne bathymetric data of the shallow sea area of Danzhou (partial) before processing. (**d**) shows the visual effect diagram of the airborne bathymetric data of the shallow sea area of Danzhou (partial) after processing. The diagram is obtained by performing elevation rendering on the point cloud data.

As demonstrated in the figure above, even at extremely low sampling rates, the DFPS algorithm demonstrates a robust capability to preserve global features. By comparing the processing results before and after using the Wufahai data (as shown in Figure 7a,b) and the local data from the Danzhou shallow sea area (as shown in Figure 7c,d), it is evident that edge terrain features and relatively flat central areas with micro-topographic height variations have been effectively retained. Furthermore, by eliminating a significant amount of redundant data caused by heading overlap and side overlap, the overall density of the point cloud becomes more consistent, thereby reducing the computational burden associated with large-scale data processing and large-scale point cloud visualization. Due to the vast amount of data in airborne point clouds and the substantial redundancy caused by heading and side overlaps, directly processing high-density point clouds would impose an immense computational burden. Appropriate downsampling not only removes redundant data caused by swath overlap and reduces point cloud density to enhance processing speed but also smooths the point cloud data, standardizes its density, and suppresses point cloud layers to improve modeling accuracy. The sampled data can serve as high-quality input for subsequent point cloud data processing. For instance, the processing of airborne blue–green laser bathymetry data from Wuhua Sea (comprising 6,091,468 points) took only 43 s, further illustrating the efficiency of DFPS in handling large-scale data.

#### 3.2.2. Experiments on Land Survey Data

**Figure 8:**
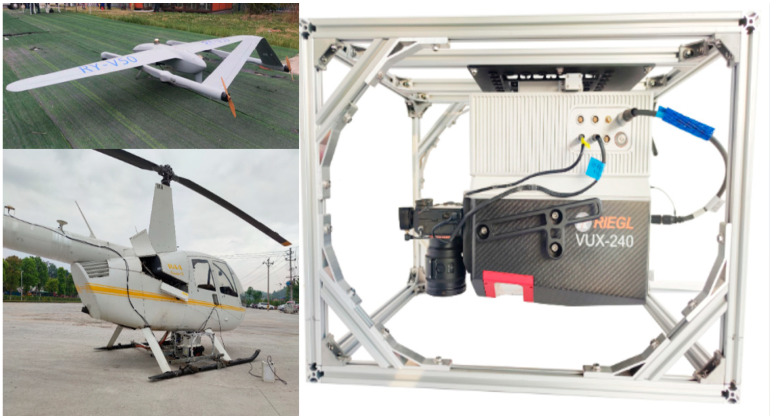
VSurs-ARL Airborne Laser Radar System Product Diagram.

The land survey data were obtained by the VSurs-ARL airborne LiDAR system of Qingdao Xiushan Mobile Measurement Co., Ltd. (Qingdao, China) for several local areas of the campus of Shandong University of Science and Technology. The VSurs-ARL airborne LiDAR system is a brand-new high-end LiDAR system with an ultra-long measurement range, which is mounted on light manned aircraft, unmanned helicopters, vertical take-off and landing fixed-wing aircraft, and multi-rotor unmanned aerial vehicle platforms. It is equipped with a full-frame mirrorless camera and a high-precision MEMS integrated navigation system, making it highly suitable for applications in strip mapping scenarios with high point density requirements, such as power line inspection and corridor mapping. Through high-precision scanning, more comprehensive and detailed original data can be acquired. After downsampling and preprocessing, the data can serve as more representative input for subsequent point cloud semantic segmentation and target recognition tasks in the region. Table 5 provides the relevant detailed parameter information of the VSurs-ARL airborne LiDAR system and Figure 8 presents the product images of the VSurs-ARL Airborne Laser Radar System.

**Figure 9:**
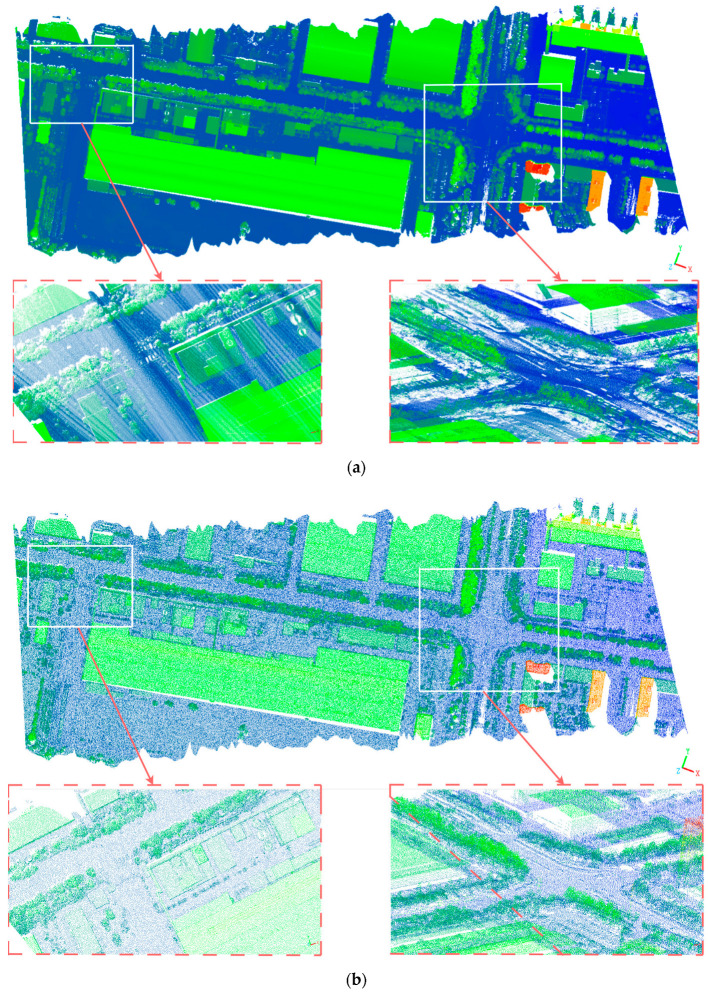
(**a**) shows the pre-processed visual effect diagram of the airborne laser point cloud data of the campus of Shandong University of Science and Technology, Shandong Province, China (a partial view). (**b**) shows the post-processed visual diagram of the airborne laser point cloud data of the campus of Shandong University of Science and Technology (a partial view). The diagram is obtained by performing elevation rendering on the point cloud data.

Figure 9 shows the data of the campus of Shandong University of Science and Technology (a partial view), which contains 8,492,248 points. Through the DFPS algorithm, a 5% sampling rate downsampling was performed, and the comparison effect diagram of the global and local visual observation is obtained:

Figure 9 provides a comparative analysis of pre-processed and post-processed airborne survey data from Shandong University of Science and Technology, China (partial study area). The results demonstrate that under low sampling rate (at 5%) conditions, the DFPS algorithm effectively preserves critical geospatial features, including vegetation distribution, road inflection points, and terrain–object boundary characteristics, as visually verifiable through the comparative visualization. Simultaneously, the DFPS algorithm achieves substantial noise suppression and successfully eliminates interference from dynamic objects (e.g., vehicular movements). Further examination of the lower-left subfigure in panel (a) reveals that the raw data exhibit pronounced striation artifacts originating from the LiDAR profile scanning mechanism, which introduces both data redundancy and adverse impacts on computational efficiency and visual interpretability during downstream processing. In contrast, the corresponding subfigure in panel (b) demonstrates the complete elimination of striation patterns through the implementation of DFPS, along with a more homogenized density distribution, higher quality, and more concise input point cloud, thus creating optimized input conditions for subsequent spatial data processing workflows, such as point cloud semantic segmentation and target recognition.

#### 3.2.3. Quantitative Analysis of Sampling Effects

Section 3.2.1 and Section 3.2.2 have preliminarily demonstrated through visual inspection that DFPS exhibits strong global feature retention capabilities, even at low sampling rates. To enhance the rigor and persuasiveness of this claim, we conduct a quantitative analysis of DFPS’s sampling performance. Point cloud downsampling aims to reduce data volume while preserving the original geometric characteristics. However, due to varying objectives—such as improving computational efficiency or enabling comprehensive feature extraction—a universally consistent metric for quantifying feature preservation is lacking. The chamfer distance addresses this challenge by measuring overall shape discrepancies between two point clouds through a bidirectional nearest neighbor search (the calculation formula is as follows [42]), thereby symmetrically quantifying differences.

$$d_{CD}\left(S_{1},S_{2}\right)=\frac{1}{S_{1}}\sum_{x\in S_{1}}\underset{y\in S_{2}}{\min}\;∥x-y{∥}_{2}^{2}+\frac{1}{S_{2}}\sum_{y\in S_{2}}\underset{x\in S_{1}}{\min}\;∥x-y{∥}_{2}^{2}$$

Here, $S_{1}\;$ and $S_{2}$ represent two point cloud datasets that need to calculate the Chamfer distance. The approach effectively mitigates the bias inherent in one-way distance measurements. Notably, the bidirectional computation ensures that the sparsity of the downsampled point cloud does not distort the evaluation results, unlike one-way distance methods. Therefore, we adopt the Chamfer distance as our evaluation metric.

In Section 1, it is mentioned that the farthest-point sampling algorithm, which has demonstrated excellent performance in preserving global features, has gained widespread recognition and has been incorporated into various neural networks. To demonstrate that the DFPS algorithm maintains a comparable level of global feature retention as the FPS algorithm, it suffices to show that the Chamfer distance between point clouds processed by the DFPS and FPS algorithms remains minimal. To enhance the credibility of the verification, the data used in this section are derived from a partial view of the campus of Shandong University of Science and Technology. The sampling rate starts at 20% and decreases gradually by 2.5% in each step until reaching 2.5%. The experiments include voxel downsampling and uniform downsampling as control methods for comparative analysis.

From Table 6, it is evident that DFPS sampling achieves results closer to farthest-point sampling when compared to voxel downsampling and uniform downsampling. This similarity in performance holds true both under medium retention conditions (such as 20% sampling rate) and low retention conditions (such as 2.5% sampling rate). When combined with the visual comparisons presented in Section 3.2.1 and Section 3.2.2, these findings strongly support the conclusion that DFPS is an efficient downsampling algorithm, particularly well-suited for global feature extraction from large-scale point cloud data.

## 4. Conclusions

This paper addresses the issues of excessive redundant points in large-scale laser point cloud data processing, which negatively impact the efficiency of subsequent point cloud data processing. Additionally, it highlights the inability of traditional downsampling algorithms to adequately preserve global features. To tackle these challenges, a DFPS (Dynamic Farthest-Point Sampling) downsampling algorithm based on an adaptive hierarchical grid partitioning mechanism is proposed. Through experimental validation, DFPS significantly enhances the algorithm’s running speed while ensuring the integrity of both global and local features of the point cloud. This provides a novel technical approach for large-scale laser point cloud data processing under resource-constrained environments.

### 4.1. Research Summary

The DFPS algorithm incorporates an adaptive multi-level grid partitioning mechanism that synergistically combines dynamic recursive sampling logic with farthest-point sampling principles. Through recursive subdivision of point clouds into computational resource-optimized grid units and implementation of a multithreaded parallel acceleration architecture, the system achieves substantial computational efficiency enhancements. This approach resolves the serialization bottleneck inherent in conventional FPS iterative processes while reducing constructor invocations to optimize memory utilization, thereby achieving balanced workload distribution and refined memory access patterns. Comparative experiments reveal that DFPS demonstrates order-of-magnitude performance enhancements ranging from ~10× to ~60× compared to a non-adaptive hierarchical grid partitioning mechanism and a multi-thread-only optimized variant, with acceleration effects becoming progressively pronounced at lower sampling rates. The adaptive grid architecture enables DFPS to maintain significant processing speed advantages across point clouds spanning three orders of magnitude (10^3^–10^6^ points). On an Intel i7-9750H platform, DFPS reduces processing latency for million-point clouds from 161,665 s (baseline FPS at 12.5% sampling) to 71.64 s (2200× acceleration), with further efficiency gains observed at reduced sampling rates: 35,060 s (baseline FPS at 3.125%) versus 3.78 s (9278× acceleration). In addition to its high processing efficiency, DFPS exhibits robust global feature retention and effective redundant point elimination capabilities. By employing visual observation and quantitative analysis (with the chamfer distance as the measurement index), it is evident that DFPS can effectively preserve global feature information even at low sampling rates. It excels in retaining micro-topography details while efficiently eliminating redundant points, thereby providing high-quality input for subsequent point cloud data processing and computations. Furthermore, leveraging a lightweight multithreaded design on the CPU, DFPS operates independently of GPU heterogeneous computing, thus avoiding reliance on specialized frameworks such as CUDA. This enables seamless deployment in resource-constrained environments, including airborne systems and mobile devices, making it especially suitable for the downsampling of large-scale laser depth point clouds following stitching.

### 4.2. Application Prospects

The DFPS algorithm has been empirically validated for computational efficiency and operational robustness. Its superior capability in preserving global structural features, coupled with high-throughput processing performance, establishes it as a general-purpose solution for large-scale point cloud datasets. This methodology effectively addresses critical challenges, including redundant data elimination and computational overhead reduction in downstream processing pipelines. Furthermore, the integration of adaptive dynamic sampling with multi-scale feature retention mechanisms demonstrates significant potential for diverse engineering applications.

1. High-Precision Point Cloud Processing Applications

DFPS exhibits distinctive capabilities in precision-critical operations, including point cloud registration, semantic segmentation, and object classification. Conventional registration methodologies suffer from accuracy degradation induced by heterogeneous feature space distributions stemming from density variations. DFPS mitigates this limitation by establishing density-uniform point distributions, thereby enhancing alignment precision in overlapping regions. For semantic segmentation tasks, the algorithm’s adaptive hierarchical grid partitioning mechanism ensures comprehensive retention of both global topological characteristics and local geometric minutiae during redundancy removal. This dual-scale preservation property enables deep-learning frameworks to achieve enhanced discriminative performance on fine-grained features, particularly in texture-deficient regions such as road boundaries and foliage edges. As neural network-based point cloud analysis remains an active research frontier, DFPS serves as a preprocessing module that optimally balances computational efficiency with semantic consistency for the algorithm based on the deep-learning method.

2. Hierarchical Multi-Resolution Management for Ultra-Scale Point Cloud

The advancement of laser scanning hardware has precipitated exponential growth in point cloud data volumes. Post-stitching processing of bathymetric point clouds in large-scale maritime environments routinely generates datasets exceeding hundreds of millions of sampling points. DFPS’s adaptive hierarchical architecture demonstrates seamless interoperability with Level of Detail (LOD) theory, enabling multi-resolution visualization and computational management of ultra-scale point clouds through GPU-accelerated processing pipelines. Integrated with a viewpoint-adaptive resolution control mechanism, the algorithm dynamically adjusts sampling densities in response to observational proximity thresholds. Spatially varying sampling strategies across resolution hierarchies ensure optimal preservation of global geometric features during rendering while maintaining perceptual fidelity, thereby substantially mitigating computational overhead in DFPS-LOD integrated systems. This framework implements tiered data streaming: low-resolution proxies facilitate real-time interaction on mobile/web platforms, whereas cloud-hosted full-resolution datasets empower semantic parsing of navigation-critical features, including reef formations and submerged structures. The DFPS-LOD synergy consequently establishes a paradigm-shifting solution to storage-computation-visualization constraints inherent in large-scale digital twin ecosystems for coastal cities and oceanic environments.

## Figures and Tables

**Table 1:** Speed comparison of three downsampling methods FPS, FPS_After, and DFPS at a sampling rate of 12.5%, with time unit being milliseconds.

Point Number	FPS	FPS_After	DFPS
2^9^	39.7925	0.4393	0.3829
2^10^	126.098	1.3916	0.9066
2^11^	515.368	2.7296	1.1822
2^12^	2192.76	10.7343	3.7438
2^13^	8535.64	43.2526	12.5042
2^14^	34,847.1	180.454	32.4967
2^15^	149,151	679.762	74.1469
2^16^	528,648	2694.88	323.329
2^17^	2,454,130	10,855	1054.55
2^18^	9,877,745	44,678.7	4668.7
2^19^	39,324,980	184,931	16,860
2^20^	161,664,989	779,888	71,644.4

**Table 2:** Speed comparison of three downsampling methods FPS, FPS_After, and DFPS at a sampling rate of 6.25%, with time unit being milliseconds.

Point Number	FPS	FPS_After	DFPS
2^9^	18.8918	0.1229	0.2426
2^10^	63.4278	0.7107	0.5581
2^11^	265.346	1.4114	0.5931
2^12^	1250.55	5.7015	1.4842
2^13^	4434.93	21.3298	5.5157
2^14^	17,299	85.0292	11.1254
2^15^	67,972	340.207	25.5064
2^16^	280,120	1363.86	79.0694
2^17^	1,068,414	5367.01	343.057
2^18^	4,252,287	22,214.7	1160.75
2^19^	16,966,628	90,707	4607.86
2^20^	68,205,844.6	367,414	20,463.1

**Table 3:** Speed comparison of three downsampling methods FPS, FPS_After, and DFPS at a sampling rate of 3.125%, with time unit being milliseconds.

Point Number	FPS	FPS_After	DFPS
2^9^	8.8215	0.0805	0.2313
2^10^	32.6853	0.2675	0.3976
2^11^	139.207	0.9026	0.4264
2^12^	737.607	3.3963	1.4083
2^13^	2218.14	11.0993	2.1371
2^14^	8331.41	42.6509	6.0818
2^15^	33,034	167.807	22.1335
2^16^	131,886	662.188	27.9496
2^17^	537,008	2603.29	84.4039
2^18^	2,158,772	10,730.7	266.29
2^19^	8,656,676	45,069.6	969.683
2^20^	35,059,537	182,743	3784.96

**Table 4:** Introduction to the detailed parameter information for the MAPPER-20kU unmanned aerial vehicle-mounted water and land detection laser radar.

Parameters	Indexes
Laser Wavelength	532 cm and 1064 cm
Laser Measurement Rate	≥20 kHz
Laser Scanning Rate	1200 rpm
Scanning Method	Oval Scanning Pattern
Swath Width	Total Field of View (FOV): 40°
Data Acquisition Mode	Full-waveform Sampling
Ground Point Density	15 points/m^2^ (at 100 m flight altitude & 20 m/s flight speed)
Maximum Detection Depth	≥2 × Secchi Disk Depth
Minimum Detection Depth	0.25 m
Bathymetric Accuracy (RMSE)	0.15 m
Vertical Positioning Accuracy	≤0.15 m
Horizontal Positioning Accuracy	≤0.4 m

**Table 5:** Introduction to the detailed parameter information for the VSurs-ARL airborne LiDAR system.

Project	Parameters	Indexes
Overall System Indicators	Positioning Accuracy	10 cm
	Weight	9 kg
Scanner (Riegl VUX-120)	Measurement Range	~5–1430 m
	Precision	0.05 cm
	Field Angle	±50° (100°)
	Single Frequency	1.8 million points/s
	Linear Frequency	400 HZ
	Vertical Field of View	Inclined forward by 10° Vertically downward Inclined backward by 10°
Compass Navigation System (SPAN-μIMU)	Positioning Accuracy (RMS)	0.01/0.02 m
	Attitude Accuracy (RMS)	0.005
	Azimuth Accuracy (RMS)	0.009
	Positioning Form	All system
	Frequency	200 HZ
Camera System (Digital Camera Optional)	Frame rate	9504 × 6336
	Resolution	1 Fps

**Table 6:** The quantitative analysis test table for sampling effectiveness of the DFPS algorithm uses the data sampled by the FPS algorithm as the benchmark. It calculates the chamfer distance for the point cloud data processed by the DFPS algorithm, voxel downsampling, and uniform downsampling with sampling rates ranging from 2.5% to 20%.

	Sampling Rate	DFPS	Uniform	Voxel
FPS	2.5%	0.7079	3.1546	3.1810
	5%	0.9732	1.8518	2.1822
	7.5%	0.6264	1.9775	1.9798
	10%	0.4894	2.0834	2.0843
	12.5%	0.6624	2.1870	2.1901
	15%	0.9078	3.0870	3.1107
	17.5%	0.7108	2.1118	2.1197
	20%	0.4020	1.8339	1.8474

## Data Availability

Data available on request due to restrictions.
